# An alternative model in the provision of CPAP in sleep apnea: a comparative cost analysis

**DOI:** 10.1186/s12913-021-06474-5

**Published:** 2021-05-18

**Authors:** Demetrio Gonzalez-Vergara, Sergio Marquez-Pelaez, Jose David Alfonso-Arias, Julia Perez-Ramos, Jose Luis Rojas-Box, Manuel Aumesquet-Nosea

**Affiliations:** 1 Unidad Médico-Quirúrgica de Enfermedades Respiratorias, Hospital Universitario Virgen del Rocío, Avda. Manuel Siurot, s/n, 41013 Seville, Spain; 2 Agencia de Evaluación de Tecnologías Sanitarias de Andalucía, Seville, Spain; 3 Agencia Sanitaria Bajo Guadalquivir, Hospital de Alta Resolución de Constantina, Seville, Spain

**Keywords:** Apnea-hypopnea syndrome during sleep, CPAP, Costs, Satisfaction

## Abstract

**Background:**

To conduct a pilot study on an alternative model for the provision of respiratory therapies in sleep apnea-hypopnea syndrome (SAHS) by internalizing the service with the purchase, monitoring and control of continuous positive airway pressure (CPAP) equipment by the hospital.

**Methods:**

An observational, prospective pilot study of comparative cost analysis by internalizing the service to include all patients up to a budget limit of 5000 euros. The cost of internalizing the service included the acquisition of CPAP equipment and all the necessary accessories in addition to the nursing days necessary to track the patients. Patient satisfaction was assessed by a survey of the hospital service.

**Results:**

Twenty-one patients with 23,046 patient-days of follow-up were included. The cost of the internalized system was 6825.11 €. The cost of the outsourced system over the same period would have been 22,781.18 €; thus, the direct saving was 15,956.07 €. The cost per device per day of the internalized system was 0.30 € versus the 0.99 € that the outsourced system would have cost during the study period. In the satisfaction survey, 12 (70.6%) patients indicated that they preferred the service of the hospital over that of the external company. No patient preferred the outsourced system.

**Conclusions:**

The internalization of CPAP service represents significant cost savings from a hospital perspective and an improvement in patients’ perceptions of the quality of service.

**Supplementary Information:**

The online version contains supplementary material available at 10.1186/s12913-021-06474-5.

## Introduction

Sleep apnea-hypopnea syndrome (SAHS) is a common disorder with a high prevalence in the general population and is associated with well-recognized detrimental health effects [1]. In this context, continuous positive airway pressure treatment (CPAP) has achieved excellent therapeutic efficacy with notable consequences for patients [2]. Notably, this disorder and its treatment are also associated with an increase in the economic burden on the health system [3]. For this reason, initiatives are needed that reduce costs and thus make a health system more sustainable.

The cost studies associated with SAHS have focused mainly on reducing the cost derived from the consequences of the disease, such as traffic accidents or comorbidities [4], and on the cost-effectiveness of different diagnostic methods [5]. However, despite studies showing the cost-effectiveness of CPAP treatment [6], the costs of the different options for providing CPAP to a large population have been less consistently evaluated. In Spain and in most other European countries, the existing model is based on outsourcing the service, with a company outside the healthcare system being assigned to provide home respiratory services, including CPAP treatment. However, the cost-effectiveness of this form of organization compared to other, non-outsourced models has not been evaluated.

The objective of this work is to carry out a pilot study on the impact on direct costs of a new model of CPAP treatment of patients with SAHS by internalizing the service with the purchase of CPAP equipment and control of therapy by the hospital itself. The working hypothesis is that the internalization of the service implies a significant decrease in costs without altering the patients’ perception of the quality of the healthcare provision.

## Methods

### Study setting

This observational, prospective study is a comparative analysis of the costs of home CPAP service in patients diagnosed with SAHS. The contracting of outsourced services in the usual practice in Spain. In this traditional outsourced system, this contracting is done by setting a price per type of home therapy, generating an estimated cost per patient and day. This calculated cost includes the cost of the devices and all the necessary support of personnel and consumables to be able to provide the service properly. After a theoretical estimate of the potential cost savings, the center decided to invest in a pilot study on the cost of health care for a group of patients with SAHS receiving CPAP treatment. This experience consisted of a change in the healthcare model to internalize the service to include all possible patients until a budget limit of € 5000 was reached for the year 2013, which limited the number of patients evaluated.

### Procedures

During the study period included patients were changed from the usual outsourced system to a new system in which they were controlled by an internal service completely dependent on the health center. During this period new CPAP patients were also directly initiated in the new internalized system. During the internalization procedure, the healthcare center was in charge of purchasing the CPAP devices as well as all the necessary accessory materials. In addition, the staff of the hospital center followed up on the therapy. In the new internalized system, patients were cared for by a nurse, who explained the treatment to them, resolved doubts, and kept track of patients for incidents. All the personnel implicated in the care of these patients were experienced qualified nurses who received a proper training before the initiation of the study. The periodicity of the patients’ regular follow-up visits did not change with the internalized system. Therefore, the health care provided was the same for both systems.

The included patients had to have a confirmed diagnosis of SAHS according to current recommendations [7] and be on CPAP treatment or have an indication to start this treatment [7]. Between October and December 2013, patients who attended the center because of SAHS were included and followed up until October 2016. The result variables were the difference in direct costs between the two systems and the evaluation of the patients’ perceptions of the quality of the system through a satisfaction survey.

### Cost estimations

The analysis evaluated the cost of the new internal system compared to the cost they would have incurred if they had remained in the external system. While the external company charges per device and day in a sustained manner throughout the treatment, the internalized system pays for the machines and consumables in full in a single payment when needed. For the study of costs, the perspective of the analysis was that of the health system, considering only direct health costs that included the acquisition of CPAP equipment and all the accessories (Table 1), including the necessary replacement of accessories during follow-up. Each CPAP came with its own tubing, so the necessary tubing had to be replaced at follow-up in case of deterioration. In addition, the cost of the nursing days necessary to carry out the follow-up of all the included patients was imputed. A microcost approach was used since it allows an accurate evaluation of the costs of health interventions and has proven to be particularly useful in estimating the cost of new interventions and the true cost to the health system [8]. The analysis was based on a detailed description by each patient of the activity carried out to identify and assess each assigned resource. The tasks and activities to be carried out, the time dedicated by the professional and the consumables necessary to carry out each task were defined. On the other hand, the allocable structure costs (management, administration and physical space for consultation) and the amount of supplies (electricity, water, telephone and thermal conditioning) were excluded from the analysis. After identifying the amounts of resources to be used, prices obtained from hospital billing were assigned if they were available; otherwise, supplier companies were consulted [9]. The costs included in the calculations are summarized in Table 1. The cost of the CPAP and the cost of other necessary equipment were directly assigned without annualization, considering the 5-year life of CPAP devices. The nursing workday was calculated according to the salary table of the collective agreement in force at the time for the Bajo Guadalquivir Public Health Agency.

**Table 1:** Direct costs included in the analysis

Concept	Cost ^a^	Number used during follow-up	Total cost during follow-up
CPAP	151.68 €	21	3185.28 €
Nasal mask	35.48 €	45	1596.60 €
Nasal gel mask	56.00 €	3	168.00 €
Oronasal mask	93.78 €	5	468.90 €
Humidifier	94.27 €	4	377.08 €
Tubes	12.63 €	3	37.89 €
Accessory mask	2.65 €	12	31.80 €
Filter	5.50 €	7	38.50 €
**Total consumable expenses**			**5904.05 €**

^a^ Retail price with value-added tax included

The resulting cost of the internalized service was compared with an estimate of what costs would have been entailed if the service had continued to be externalized. To estimate the calculation of spending with the outsourced system, due to changes in the rental system rate during the follow-up period, the number of days that each patient remained in the study was divided into three periods. Thus, rate 1 of € 1.10 per day was applied from the date of entry to the study until January 31, 2014; rate 2 of € 0.86 per day was applied from February 1 until July 6, 2014; and finally, rate 3 of € 0.65 per day was applied from July 7 onwards.

### Patients satisfaction evaluation

For the study of patient satisfaction, a simple satisfaction survey specifically created for this project (supplementary file) was carried out on the service provided by the hospital; Patients were asked 3 questions: their degree of satisfaction with the explanations and the treatment received with each system (with 4 possible answers: very satisfied, satisfied, dissatisfied, and very dissatisfied), the quality of the service provided with each system (with 4 possible answers: very good, good, bad, and very bad) and who they believed offered a better service (with 4 possible answers: the external company, the center, both equally and not applicable). Patients who were new to CPAP treatment and had no experience with the outsourced system limited their answers to the internalized system only. This survey was anonymous, was completed by the patient in the waiting room and was delivered in a sealed envelope.

The patients signed a written informed consent agreeing to participate in the study prior to their inclusion in it for data collection with a view to subsequent analysis. There were no personal data in the database that would allow a patient to be identified.

### Analysis of data

The evaluation of the cost differences was performed by analyzing the total cost of each system during the period studied. In addition, the cost per device and day was estimated, as it was the way that external companies invoiced. To estimate the potential savings in a larger area, after evaluating the cost differences between the two systems, the cost differences if the results were applied to our entire healthcare area until 2019 were estimated.

Finally, the possible impact of an increase in the price of CPAP was estimated through a sensitivity analysis, with the price of the devices increased by 25 and 50% and the differences until 2019 estimated. Differences in the satisfaction questionnaire were studied by Chi-square or Fisher’s exact test, with a significance level of 0.05, using the IBM SPSS Statistics program (IBM Corporation, Armonk, NY) version 26.0.

## Results

During the recruitment period, 21 patients were included, of whom 4 were new cases and 17 were patients who were already receiving CPAP. The included patients were mostly male (95.23%), with a mean age of 56 years and a body mass index of 33.72 kg/m^2^. Patients who were already in treatment had an average hourly compliance of 6.6 h per day.

The total number of days accumulated by patients in the internalization system was 23,046 patient-days. The material needed during the follow-up is summarized in Table 1. The expenditure on devices and consumables for these patients was € 5904.05. When the nursing hours for the control of these 21 patients for full days counted at a cost of € 153.51 are added, this represents a total expense of € 6825.11.

The cost of having these patients undergo CPAP through the outsourced system during this period would have been € 22,781.18. Therefore, with the proposed calculations and under the assumptions made, compared to the externalized system, the internalized system means a saving of € 15,956.07 for these 21 patients. Depending on the type of patient (new or revision), the internalized system represents a saving of € 3065.38 for new patients (an average of € 766.34 per new patient) and € 12,890.69 for patients who were already in treatment (an average of € 758.27 per patient already previously diagnosed). If we transfer it to a price per device per day, which is how external companies tend to bill, the internalized system in this period would cost € 0.30 per device per day compared to the € 0.99 that the system would have cost if it had been outsourced during the study period.

**Fig. 1:**
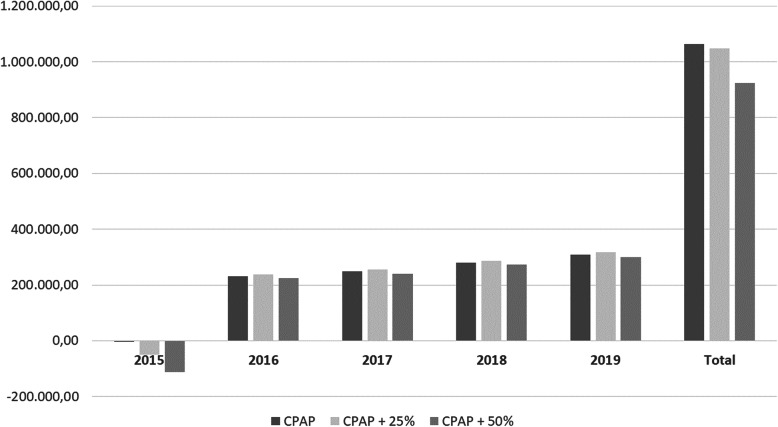
Analysis of sensitivity on budgetary impact presenting the estimated savings considering increases in the price of devices by 25 and 50%

At the time of the analysis, the sanitary area of the Bajo Guadalquivir Health Agency was made up of 3 high-resolution hospitals with a pneumology service that had an assigned population of 135,136. The estimate of the cost of the internalized system extended to this health area until 2019 is reflected in Table 2. Despite the fact that the first year would involve an investment, in subsequent years, a sustained saving of approximately 50% would be maintained. The results of the analysis with the increase in prices and the estimate until 2019 are reflected in Fig. 1. In potential scenarios in which the price of CPAP increases, the internalized system would still be cheaper than the traditional system.

**Table 2:** Budget impact extrapolating data to the entire health area including three local hospitals

	Traditional system cost	Alternative system cost	Cost difference	Difference (percentage)
Year 2015	376,073.09	379,053.01	− 2979.92	−0.79
Year 2016	414,545.37	183,882.33	230,663.04	55.64
Year 2017	456,953.36	208,362.92	248,590.44	54.40
Year 2018	503,699.69	224,542.51	279,157.18	55.42
Year 2019	555,228.16	247,632.86	307,595.30	55.40
Total	2306,499.66	1243,473.62	1,063,026.04	46.09

The results of the user satisfaction survey are summarized in Table 3. When asked which system they preferred, 12 (70.6%) patients indicated that they preferred the hospital center service over the external company, and 5 (29.4%) indicated that they found the two equal.

**Table 3:** Results of the satisfaction survey

	Internalized system results (*n* = 21)	External company results (*n* = 17)	*P* value *
Degree of satisfaction with the explanations and the treatment received.			
Very satisfied	15 (71.4%)	2 (11.7%)	< 0.001
Satisfied	6 (28.6%)	12 (70.6%)	
Dissatisfied	0	2 (11.7%)	
Very dissatisfied	0	1 (5.8%)	
Quality of service provided			
Very good	15 (71.4%)	2 (11.7%)	< 0.001
Good	6 (28.6%)	13 (76.4%)	
Bad	0	0	
Very bad	0	2 (11.7%)	
Which of the two systems offers a better service?			
External company		0 (0%)	
Internalized system		12 (70.6%)	
Both equally		5 (29.4%)	

* Calculated using the Chi-square test

## Discussion

The present work explores the impact on the direct costs of internalizing the home CPAP service in patients with SAHS. The data presented show that the internalization of the service represents a lower cost than that of the current externalized service, finding a significant cost saving and with a higher level of satisfaction perceived by the patient. Given the high prevalence of SAHS and the proven clinical efficacy of treatment with CPAP [10], the cost of this treatment marks SAHS as one of the chronic diseases with a high impact on the cost of the health system [3]. Consequently, all initiatives that provide added value in cost containment while maintaining the effectiveness and user satisfaction of the treatment should be explored.

Initially, home therapy companies were responsible for oxygen therapy. This was administered through oxygen in cylinders that were left at the patient’s home and had to be recharged periodically. This required a very specialized service and health care centers chose to outsource this service. However, one of the risks of outsourcing healthcare services, such as home respiratory therapies, is the possibility that the healthcare organization will be excluded due to certain technological changes captured by the contracted providers [11, 12]. However, in SAHS, the main treatment modality that occurs in respiratory therapies is CPAP, which does not require any type of storage or specific transport. In addition, the cost of CPAP treatments is progressively lower, and numerous models have appeared that do not need complex technical support beyond a small technical revision of the periodic device and an occasional change of mask, tubing and filters. Therefore, it seems that the need for a supplier company is becoming steadily less justifiable since the conditions that initially led to the outsourcing of home respiratory therapies have changed considerably.

In Spain, the selection of the provider of home health services is carried out through a competitive public call in which interested companies compete. Therefore, the chosen company is the one that usually has the best cost / service ratio. Once awarded, the company maintains the contract for a number of years specified in each call. Therefore, our new internalized system was compared to the cost of this winning company, which is considered the most cost-effective. Because of the selection system, vendors detail in their proposals all specificities including the personnel they use. As per Spanish regulations, this type of work must be done by a qualified trained respiratory nurse or therapist as in the hospital. Therefore, the qualification of the personnel is similar in both systems.

In our work, the gross difference in direct costs between the two systems represents a considerable avoided cost 3 years after implementation, with a notable difference calculated on the basis of total cost and cost per device per day. The differences between the two systems were expected to be detected, since an external company has to cover the costs of material and personnel and also add the cost of its organizational structure and the profit it must obtain as a company. However, the internalized system is non-profit making and therefore only has costs derived from healthcare. In the internalized system, CPAPs are purchased and used with the goal of keeping it in use throughout its useful life in each patient. These CPAPs are neither rented nor sold. This is important to keep in mind because the system must become progressively more convenient over time as the CPAP is amortized. Given the form of calculation, it is expected that the cost per device and day of the internalized system will tend to decrease over time since the cost of acquiring the CPAP is spread over the number of days that it is used. On the other hand, in the case of outsourcing, the cost per device and day approaches the figures for rates 2 and 3, which are significantly lower than those for rate 1 described in the method. In any case, the cost per device and day will always be lower in the internalized case.

One of the key reasons for choosing this type of partial economic evaluation analysis is that a change in the acquisition system does not mean an alteration in the effectiveness of the treatment, since the patient receives the same device in both forms of management and is not affected by how it is acquired. Therefore, our analysis is based on a detailed description of the activity carried out to identify and assess each assigned resource that is called the activity-based cost system [13]. This analysis is especially appropriate for estimating costs in the health field, given that the activities generators of the uses, consumption or wear of productive factors are applied to each patient depending on the specific requirements of each diagnostic-therapeutic process and each clinical condition [14].

Some authors have indicated that the calculation of costs should be carried out by annualizing the initial capital outlay for the useful life of the asset, thus obtaining the equivalent annual cost, since this automatically considers both the depreciation aspect and the opportunity cost aspect of the asset. Regarding cost of capital, in our case, the calculation has not followed this method. In the present analysis, the cost of the CPAP and the cost of other necessary equipment (mask and humidifier) have been assigned directly without annualization. This consideration overestimates the annual cost per patient of the acquisition proposal versus the rental, especially the year of acquisition of the device, in which it would be very difficult for the proposal to obtain savings compared to the outsourced daily rental system. However, this mode of accounting faithfully reflects the actual expense incurred at each time for each patient.

Our study has various methodological considerations. The cost of the internalized system may vary depending on the price agreed with different commercial vendors for the device. In our case, we believe that the price we use to carry out the calculations is more unfavorable than that obtained by private providers of respiratory therapies. Furthermore, it would be normal for prices to go down once an offer to purchase the equipment has been made through a public tender. However, these prices may vary according to the different local agreements that are reached, or even owing to pressure from the supplier companies on the CPAP commercial vendors. In this sense, the analysis provided by increasing the price of the devices by 25 and 50% provides us with information on more adverse scenarios and a comparison with the traditional outsourced system. Another consideration is that the costs of the two systems being compared and the differences between them are dynamic and change every day. When carrying out the comparative cost analysis, we must take a still photo, with positive results for the alternative system. We cannot know exactly what will happen in the future, but as we have stated, the main investment, the purchase of CPAP devices, has already been made. If we take into account an estimated useful life of 5 years, it is reasonable to posit that savings will increase in the coming years, and the larger the reference area is, the greater the impact will be. Another consideration is that our calculations do not include indirect costs derived from the storage of the material since the number of patients is small. If the calculation is scaled to a higher service, this cost will likely need to be added. However, with the results of our analysis, it is expected that the internalized option will continue to be cheaper. Our study measures the cost of providing CPAP during 3 years of follow-up. The cost of this treatment is constant regardless of how long you have been using CPAP treatment. In fact, in the outsourced system, companies charge the same for each patient, whether new or old. However, since it is true that new patients may have a higher cost at the beginning for reasons of adaptation to CPAP, in our work we wanted to unite both types of patients to more closely resemble the real life of the outsourced system. Another aspect worth commenting is about the cost of storage. In real-life high-volume sleep apnea services, inventory ordering, management, and storage is not a trivial issue, and there may be costs associated with it. Some hospitals may already have storage rooms for this need but other would need to hire it. Since ours is a pilot evaluation, we did not have the need to compute this cost. Additionally, adherence to treatment is important. In this analysis we have not included possible differences in adherence to treatment between the two systems because the cost of the service is the same, whether they use it or not. However, there could be differences in adherence with a clinical impact that have not been explored. Finally, there may be differences between countries. Interestingly, in the case of sleep apnea treatment, most countries have an outsourced system similar to the Spanish one, but costs can change between countries. Therefore, these results can only be applied to Spain and the differences in other countries are to be explored.

Although a change in the acquisition system does not imply an alteration in the effectiveness of the treatment, the evaluation of the quality of the system through satisfaction questionnaires proposed for the control of the patients by the clinicians involved does show information in this regard, as the patients perceived an improvement with the new internalized system. Therefore, in addition to significantly reducing costs, this change produces an improvement in the patients’ perceptions of the quality of service. When monitoring is performed by the hospital itself, with a nursing team linked to the pulmonology service, technical and clinical monitoring of the therapy can be carried out, and duplication can be avoided. In a study carried out by Antic et al. [15], it was found that follow-up by a specialized nursing team with the occasional help of a pulmonologist is as effective as exclusive follow-up by a pulmonologist in these patients, which can translate into savings in consultations and unnecessary medical expenses, although, being conservative, we have not considered this aspect in our cost analysis.

In short, the comparative analysis of costs carried out indicates that the internalization of the service, that is, the acquisition of CPAP equipment owned by the hospital and the monitoring and maintenance of the therapy from the hospital center, represents significant savings from the perspective of the hospital and an improvement in the patients’ perceptions of the quality of service.

## Supplementary Information

**Additional file 1.**

## Data Availability

The datasets used and/or analyzed during the current study are available from the corresponding author on reasonable request.

## Abbreviations

CPAP: Continuous positive airway pressure
SAHS: Sleep apnea-hypopnea syndrome
