# Supplementary material for: An alternative model in the provision of CPAP in sleep apnea: a comparative cost analysis
Source: BMC Health Serv Res. 2021 May 18;21:469. doi: 10.1186/s12913-021-06474-5 (PMC8130106; doi:10.1186/s12913-021-06474-5)
Supplement: Supplementary file 1 — Additional file 1. [file 12913_2021_6474_MOESM1_ESM.pdf]

Agencia Sanitaria Bajo Guadalquivir
CONSEJERÍA DE IGUALDAD, SALUD Y POLÍTICAS SOCIALES

Satisfaction survey of patients who have received treatment by the hospital center

		Dial with an X
Are you satisfied with the treatment and explanations received by the **nurses and nurses** of the hospital?	Very satisfied	
	Satisfied	
	Dissatisfied	
	Very dissatisfied	
	Don't know, don’t answer	

If you have had to call the **telephone service,** are you satisfied with the resolution of incidents/doubts?	Very satisfied	
	Satisfied	
	Dissatisfied	
	Very dissatisfied	
	Don't know, don’t answer	
	I haven't had to call.	

Are you satisfied with the **ease of accessing** the Pneumology medical consultation when necessary?	Very satisfied	
	Satisfied	
	Dissatisfied	
	Very dissatisfied	
	Don't know, don’t answer	
	It wasn't necessary.	

How would you value the **service provided** by the hospital based on your experience?	Very satisfied	
	Satisfied	
	Dissatisfied	
	Very dissatisfied	
	Don't know, don’t answer	

Globally, who do you think has offered you a **better service,** the supplying company or the hospital?	The supply company	
	The hospital	
	Both equally	
	Don't know, don’t answer	

Hospital de Alta Resolución Sierra Norte
C/ Dr. Larrauri, s/n. 41450 Constantina (Sevilla)
Tel. 955889043 - Fax. 955889105
www.juntadeandalucia.es/epsbg
